# Improved Interface Charge Transfer and Redistribution in CuO‐CoOOH p‐n Heterojunction Nanoarray Electrocatalyst for Enhanced Oxygen Evolution Reaction

**DOI:** 10.1002/advs.202103314

**Published:** 2021-10-12

**Authors:** Jing Hu, Adel Al‐Salihy, Jing Wang, Xue Li, Yanfei Fu, Zhonghua Li, Xijiang Han, Bo Song, Ping Xu

**Affiliations:** ^1^ MIIT Key Laboratory of Critical Materials Technology for New Energy Conversion and Storage School of Chemistry and Chemical Engineering Harbin Institute of Technology Harbin 150001 P. R. China; ^2^ National Key Laboratory of Science and Technology on Advanced Composites in Special Environments Harbin Institute of Technology Harbin 150001 P. R. China

**Keywords:** electrocatalysis, interface charge transfer and redistribution, oxygen evolution reaction, p‐n heterojunction

## Abstract

Electron density modulation is of great importance in an attempt to achieve highly active electrocatalysts for the oxygen evolution reaction (OER). Here, the successful construction of CuO@CoOOH p‐n heterojunction (i.e., p‐type CuO and n‐type CoOOH) nanoarray electrocatalyst through an in situ anodic oxidation of CuO@CoS*_x_* on copper foam is reported. The p‐n heterojunction can remarkably modify the electronic properties of the space‐charge region and facilitate the electron transfer. Moreover, in situ Raman study reveals the generation of SO_4_^2−^ from CoS*_x_* oxidation, and electron cloud density distribution and density functional theory calculation suggest that surface‐adsorbed SO_4_^2−^ can facilitate the OER process by enhancing the adsorption of OH^−^. The positively charged CoOOH in the space‐charge region can significantly enhance the OER activity. As a result, the CuO@CoOOH p‐n heterojunction shows significantly enhanced OER performance with a low overpotential of 186 mV to afford a current density of 10 mA cm^−2^. The successful preparation of a large scale (14 × 25 cm^2^) sample demonstrates the possibility of promoting the catalyst to industrial‐scale production. This study offers new insights into the design and fabrication of non‐noble metal‐based p‐n heterojunction electrocatalysts as effective catalytic materials for energy storage and conversion.

## Graphical abstract

CuO@CoOOH p‐n heterojunction nanoarray electrocatalyst with surface‐adsorbed SO_4_^2−^ provides enhanced electrochemical oxygen evolution reaction activity and long‐term stability in alkaline media, due to modified electronic properties of the space‐charge region and facilitated electron transfer.

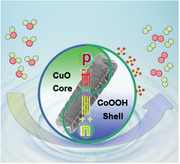

## 1 Introduction

Electrocatalytic water splitting has been considered as one of the most promising strategies for the sustainable production of hydrogen.^[^ ^1^ ^]^ However, the efficiency of this process is limited by the anodic oxygen evolution reaction (OER) because of its sluggish four‐electron kinetics and high energy barrier for O═O bond formation.^[^ ^2^ ^]^ Although benchmark RuO_2_ and IrO_2_ are known as efficient catalysts for the OER process, their high cost and poor stability limit their scale‐up application for water splitting.^[^ ^3^ ^]^ Various non‐noble metal based OER electrocatalysts based on first‐row transition metals (TMs), including Mn, Fe, Co, and Ni, have been developed.^[^ ^4^ ^]^ Cu is the second least‐expensive element among these first‐row metals and shows the highest reversibility. However, efficient Cu‐based OER electrocatalysts are far less common than other TM‐derived compounds. Up to now, CuO‐based materials may be used as Cu‐containing electrocatalysts for the OER process but are rarely applied because they suffer from inferior catalytic activity.^[^ ^5^ ^]^

Construction of nanoalloys or heterostructures is a commonly used strategy to improve the OER performance of Cu‐based catalysts.^[^ ^6^ ^]^ Cu and Co have been demonstrated to be a good couple for the OER. For example, Yang et al. comprehensively investigated the doping effect of Fe, Co, and Ni by comparing the performance of various doped CuO OER electrocatalysts.^[^ ^7^ ^]^ It was found that the CuO*_x_* nanoarray film doped with 0.30% Co (atomic ratio) could provide the optimum OER performance, requiring an overpotential of 290 mV at 10 mA cm^−2^. Besides, Sun et al. found that the activities of CuCo‐based Mott–Schottky electrocatalysts could be enhanced due to abundant active sites and increased electrical conductivity based on their potential synergetic effect.^[^ ^8^ ^]^

Therefore, in recent years, increasing attention has been paid to the construction of heterojunction in electrocatalysts for water splitting owing to the synergistic effects in achieving higher specific surface area and reduced activation energy.^[^ ^9^ ^]^ With this regard, significant progress has been made in p‐n heterojunction electrocatalysts thanks to its capability of adjusting the electronic structure.^[^ ^10^ ^]^ A p‐n heterojunction can be formed at the interface when a p‐type semiconductor meets a n‐type semiconductor, and the electronic structures of the corresponding semiconductors thus will reach a thermal equilibrium state, leading to a strong built‐in field at the p‐n heterojunction interface.^[^ ^11^ ^]^ In addition, high‐energy interfacial structures can facilitate the adsorption of targeted ions on the surface and promote the charge‐transfer process, contributing to the enhanced catalytic activity.^[^ ^12^ ^]^ Besides the construction of heterostructure, surface adsorption on electrocatalyst is another crucial factor that influences the electrocatalytic performance.^[^ ^13^ ^]^ It was reported that specific adsorption on the catalyst surface can affect the electrochemical reaction rate,^[^ ^14^ ^]^ alter the surface charges,^[^ ^15^ ^]^ hydrophilicity,^[^ ^16^ ^]^ and free energy.^[^ ^17^ ^]^

It has been mentioned in most studies that copper foam (CF) can be used as electrode materials (carrier materials), catalyst carriers, and electromagnetic shielding materials.^[^ ^18^ ^]^ Herein, copper foam is used as the Cu source and growth sites to in situ grow CuO nanoarrays for electrocatalysis, and a monolithic CuO@CoOOH p‐n heterojunction has been constructed on 3D conductive CF for enhanced OER electrocatalysis in alkaline media through controlled solvothermal route and in situ anodic oxidation process. Experimental and theoretical simulation results indicate that in this p‐n heterojunction, the positive charged n‐type CoOOH becomes more conducive to the adsorption of OH^−^ and the adsorbed sulfate ions released during the in‐situ anodic oxidation process also contributes to the electrocatalytic activity. Exceptionally enhancement in electrocatalytic OER activity and robust stability can be obtained on the CuO@CoOOH p‐n heterojunction, requiring a low overpotential of 186 mV to achieve a current density of 10 mA cm^−2^ and a small Tafel slope of 51.7 mV dec^−1^ in 1.0 m KOH solution. More importantly, our preparation methodology enables a scaled‐up electrode fabrication (14 × 25 cm^2^) under laboratory conditions, showing the feasibility of its mass production. This work provides a new pathway for designing and fabricating large‐scale and highly active p‐n heterojunction electrocatalysts for water splitting.

## 2 Results and Discussion

**Figure 1:**
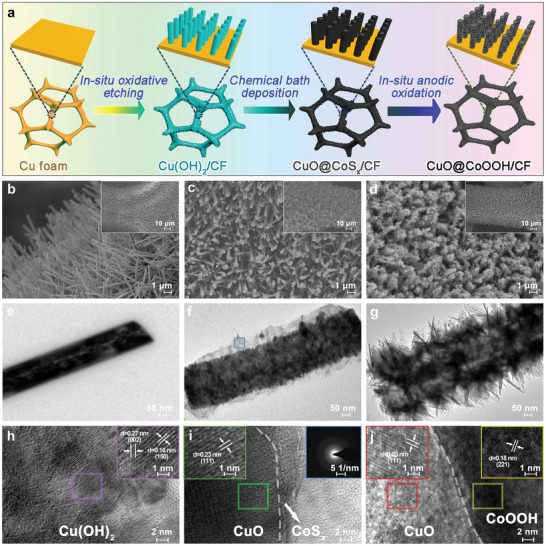
a) Schematic illustration for preparing CuO@CoOOH on copper foam (CF) in three steps: a controlled in situ oxidative etching method to achieve Cu(OH)_2_ nanoarrays, chemical bath deposition method to prepare CuO@CoS*_x_* nanoarrays, and a subsequent in situ anodic oxidation process to obtain CuO@CoOOH nanoarray heterostructures. SEM, TEM, and HRTEM images of the as‐prepared b,e,h) Cu(OH)_2_, c,f,i) CuO@CoS*_x_* heterostructures, and d,g,j) CuO@CoOOH heterostructures.

In a typical experiment, CuO@CoOOH/CF was prepared in three steps as a self‐supported electrode (**Figure** **1a**). Controlled in situ oxidative etching of the CF could lead to Cu(OH)_2_ nanoarrays supported on the CF. Through the chemical bath deposition process in ethanol solution of cobalt nitrate and thioacetamide, a thin layer of CoS*_x_* was produced accompanied by the transformation of Cu(OH)_2_ into CuO,^[^ ^19^ ^]^ leading to core–shell CuO@CoS*_x_* nanoarrays, with the color of the material changed from blue to black. Subsequently, an in situ anodic oxidation process transformed CuO@CoS*_x_* into CuO@CoOOH, where SO_4_^2−^ ions would be generated and adsorbed on the catalyst surface. It has been reported that thermodynamic unstability often leads to surface oxidation of various metal sulfide catalysts under a relatively high potential in strong alkaline solutions during the OER.^[^ ^20^ ^]^

The morphological and structural changes during the preparation process of CuO@CoOOH/CF were observed by electron microscopy techniques. Field‐emission scanning electron microscopy (FESEM) images reveal the homogeneous growth of Cu(OH)_2_ with smooth‐faced nanowires (≈4–6 µm in length and ≈150–250 nm in diameter) on the CF (Figure 1b), and similar morphological features of the CuO nanowires are displayed in Figure S1 (Supporting Information). Transmission electron microscopy (TEM) and high‐resolution TEM (HRTEM) images also demonstrate the smooth texture and crystalline characteristics of Cu(OH)_2_ (Figure 1e,h). The interplanar distances of 0.16 and 0.27 nm can be attributed to the (150) and (002) facets of Cu(OH)_2_. After chemical bath deposition, a transparent gauze‐like CoS*_x_* thin layer (10–50 nm) was deposited over the CuO nanowires to form CuO@CoS*_x_* core–shell nanowire arrays (Figure 1c). The TEM and HRTEM images in Figure 1f,i clearly verify the presence of a core–shell structure with an obvious interface. In the core region, crystalline characteristics with an interplanar distance of 0.23 nm could be indexed to the (111) facet of CuO. In the shell region, a thin amorphous CoS*_x_* layer covers the surface of CuO. The amorphous nature of this layer is confirmed by the selected‐area electron diffraction (SAED) pattern in the inset in Figure 1i. Interestingly, the SEM and TEM images in Figure 1d,g indicate that the original gauze‐like CoS*_x_* self‐reconstructs into transparent nanosheets covering the CuO nanoarrays after subsequent anodic oxidation. In addition, no crosslinking, overlap or agglomeration appeared between the core–branch nanoarray structures because of the expansion of the diameter, which could be confirmed by the surface areas that measured by the N_2_ adsorption–desorption experiments (Figure S2, Table S1, Supporting Information). Thus, CuO@CoOOH features a relatively larger catalytic surface area. The HRTEM image in Figure 1j shows crystalline nature of the catalyst with an interplanar distance of 0.18 nm, correlating well with the (221) plane of *β*‐CoOOH (JCPDS No. 72‐2280), indicating the successful transformation from CuO@CoS*_x_* into CuO@CoOOH.

**Figure 2:**
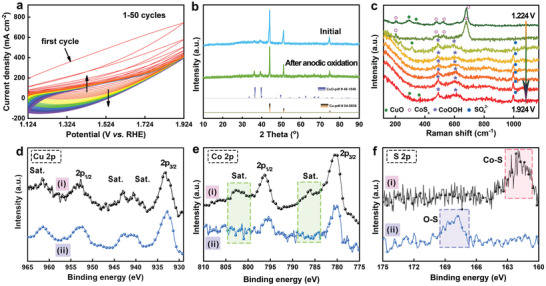
a) The 1–50 CV cycles of the anodic oxidation process of CuO@CoS*_x_*/CF in 1.0 m KOH solution. b) XRD patterns, c) in situ Raman spectra, XPS spectra of d) Cu 2p, e) Co 2p, and f) S 2p before (i) and after (ii) the anodic oxidation of CuO@CoS*_x_*/CF.

The cyclic voltammogram (CV) curves of the anodic oxidation process were measured in the potential range of 1.124–1.924 V (vs RHE) at a scan rate of 50 mV s^−1^ in 1.0 m KOH solution. As shown in **Figure** **2a**, a very strong linear dependence of the electron‐transfer process over a broad potential range could be observed during the first cycle. The current density then will be decreased considerably in subsequent CV scans. The electrochemical response gets stabilized and ultimately reaches a steady state after 50 cycles, pointing out an irreversible chemical transformation of the prepared materials.^[^ ^21^ ^]^ From the powder X‐ray diffraction (XRD) patterns (Figure 2b), only diffraction peaks of CuO and Cu are observed before and after the anodic oxidation process. To eliminate the influence of CF, the powder was scraped down and put on a glass substrate for analysis. As shown in Figure S3 (Supporting Information), only diffraction peaks of CuO can be detected, likely because of the amorphous feature or low amount of the Co‐containing species besides CuO, which also proves that the structure of CuO and Cu substrate both remained unchanged after the anodic oxidation. In addition, we further collected a potential‐dependent in situ Raman spectrum to confirm the composition and monitor the structure evolution of the catalysts during the anodic oxidation process (Figure 2c). At 1.224 V, the spectrum showed characteristic peaks of CuO and CoS*_x_*.^[^ ^22^ ^]^ As the electrode potential is systematically increased in the anodic direction to 1.429 V, the intensity of the characteristic peaks of CoS*_x_* gradually decreases, and new bands corresponding to CoOOH appeared at ≈480 and ≈600 cm^−1^.^[^ ^23^ ^]^ The appearance of these bands confirms the formation of CoOOH (from Co^2+^ to Co^3+^). Accompanying the changes in Raman bands from CoS*_x_* to CoOOH, another very interesting phenomenon is that a new peak assigned to the S—O stretching mode of SO_4_^2−^ also appears at ≈990 cm^−1^.^[^ ^24^ ^]^ There is a striking feature that as the potential increased from 1.529 to 1.929 V, the evolution of the Raman bands is stabilized and becomes irreversible during the anodic oxidation process. This reveals that CoS*_x_* will be irreversibly transformed into CoOOH during the anodic oxidation process, with the generated SO_4_^2−^ adsorbed on the material surface.

We further conducted X‐ray photoelectron spectroscopy (XPS) to investigate the electronic states of Cu, Co, S, and O in the electrocatalyst to further confirm this transformation (Figure 2d–f, Figures S4–S6, Supporting Information). The Cu 2p spectra obtained before and after the anodic oxidation are presented in Figure 2d. Besides the two major peaks attributed to Cu 2p_3/2_ and Cu 2p_1/2_, the appearance of shake‐up peaks demonstrates the existence of the Cu(II) oxidation state in CuO.^[^ ^25^ ^]^ Therefore, CuO in the samples remains intact during the anodic oxidation process. The formation of Co(III) species after the anodic oxidation process could be confirmed by the absence of multiple electron excitation satellites in the Co 2p spectrum in Figure 2e.^[^ ^26^ ^]^ In addition, the characteristic peak of the metal‐sulfide at ≈162 eV completely disappeared, and a new peak at ≈168 eV that could be indexed to SO_4_^2−^ (S—O bond) was detected (Figure 2f).^[^ ^27^ ^]^ The above results again confirm the successful fabrication of CuO@CoOOH composites via the proposed anodic oxidation method and the production of SO_4_^2−^ ions from CoS*_x_* oxidation. Considering that XPS is a surface sensitive characterization method, we further verify the structure of CuO@CoS_x_ and CuO@CoOOH by Ar^+^ etching XPS analysis (Figure S7, Supporting Information), where one can see that the elements of Co and S are mainly distributed on the surface of the sample while the Cu mostly concentrating in the interior of the sample before and after the in situ anodic oxidization process, in accordance with the previous SEM and TEM results.

**Figure 3:**
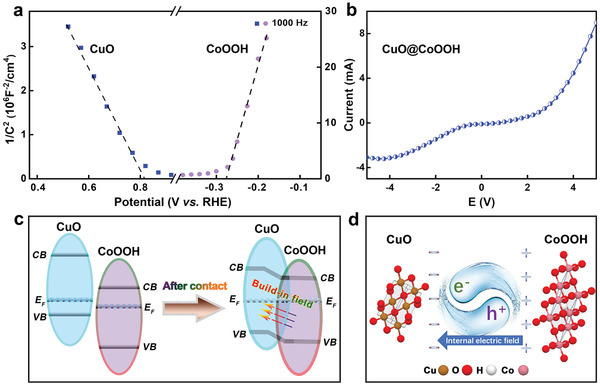
a) Mott–Schottky plots of CuO/CF and CoOOH/CF electrode, respectively, at frequency of 1000 Hz. b) LSV curves of CuO@CoOOH/CF in potential range of −5 to 5 V. c) Energy band diagram of CuO and CoOOH before and after contact. d) Schematic illustration of space‐charge region at the contact zone of p‐type CuO and n‐type CoOOH heterojunction.

Mott–Schottky curves and linear sweep voltammetry (LSV) curves were collected for a better understanding of the synergy and interactions between CuO and CoOOH. As shown in **Figure** **3a**, CuO presents a negative slope when constructing the tangent line of the longest linear part, typical for a p‐type semiconductor. By comparison, the plot of CoOOH shows a positive slope, thereby reflecting the n‐type character of this species. LSV curves were obtained over the voltage range from −5.0 to 5.0 V by clamping the flat CuO/CF electrode together with the flat CoOOH/CF electrode (Figure 3b). The composite CuO@CoOOH electrode possesses typical current asymmetric rectification effect under contact, which is due to the unidirectional mobility of electrons due to the separation of charge and hole at the interface between CuO and CoOOH, thus illustrating the existence of p‐n heterojunction induced internal electric field formed at the CuO and CoOOH interface.^[^ ^28^ ^]^ The successful construction of p‐n heterojunction contributes to the promotion of migration ability of the electrons and the formation a strong built‐in electric field.^[^ ^10b,c^ ^]^ The energy band diagrams of CuO and CoOOH before and after contact were constructed according to the above results, and the space‐charge region at the interface was also illustrated, as shown in Figure 3c,d. When the p‐type CuO comes into contact with the n‐type CoOOH, a p‐n heterojunction is formed at their interface. In this case, electrons flowing from the latter to the former and recombine with holes at the VB of CuO until the Fermi equilibrium state is achieved. This phenomenon leads to the formation of a space‐charge region at the interface, a shift in energy level from CoOOH to CuO with band bending. As a result, many active sites can be formed at CoOOH surface, promoting the transfer of OH^−^ in the alkaline electrolyte to the VB of CoOOH and enhancing its adsorption capacity, and facilitating the OER process compared to their individual counterparts.

**Figure 4:**
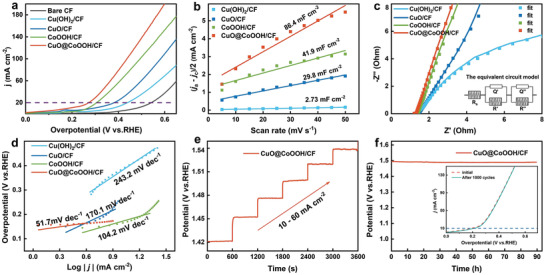
Electrocatalytic performance of various samples for OER in 1.0 m KOH. a) Linear sweep voltammetry (LSV) curves at 20 mA cm^−2^ after *iR* correction, b) electrochemical double‐layer capacitances (*C*_dl_), c) electrochemical impedance spectroscopy (inset shows the equivalent circuit used to simulate the Nyquist plots), d) Tafel plots, e) multistep chronopotentiometric curve obtained with CuO@CoOOH/CF electrode (without *iR*‐correction), and f) the long‐term stability of CuO@CoOOH/CF under a constant current density of 20 mA cm^−2^ (inset: the LSV curves before and after 1000 cycles).

Before the OER test, 50 CV cycles were applied at a scan rate of 50 mV s^−1^ to activate the catalysts and remove surface pollution.^[^ ^29^ ^]^ The OER performances of the as‐prepared samples were tested in 1.0 m KOH solution with *iR* compensation with a scan rate of 1 mV s^−1^ at room temperature. Here, the as‐prepared samples were used directly as the working electrodes, and blank CF was used as a reference. We measured the Faradaic efficiency, in situ Raman spectra, and XRD patterns at different overpotentials (Figures S8–S10, Supporting Information), which indicate that the slow current increase below 10 mA cm^−2^ was mainly from the electric double‐layer charging current (ion adsorption and migration) rather than water splitting. As the onset overpotential is near the overpotential corresponding to the current density at 20 mA cm^−2^, we compared the performances of the prepared samples at a current density of 20 mA cm^−2^. The LSV curves shown in **Figure** **4a** reveal that bare CF has negligible OER activity, and Cu(OH)_2_/CF, CuO/CF, and CoOOH/CF all exhibit limited activity with overpotentials of 440, 382, and 307 mV, respectively, to deliver a current density of 20 mA cm^−2^. Notably, CuO@CoOOH/CF affords the same current density at a much lower overpotential of 273 mV, which is at least comparable to or even better than most reported OER electrocatalysts in alkaline solutions (Table S2, Supporting Information).^[^ ^30^ ^]^ In addition, CuO@CoOOH also shows the lowest overpotential (346 mV) under high current density of 50 mA cm^−2^ compared with Cu(OH)_2_/CF (547 mV), CuO/CF (476 mV), and CoOOH/CF (385 mV), further revealing its superior OER property. The electrochemical double‐layer capacitance (*C*_dl_) of the as‐synthesized catalysts was assessed via CV method over a non‐faradaic potential range to evaluate their electrochemically active surface area (ECSA). The corresponding *C*_dl_ values of Cu(OH)_2_/CF, CuO/CF, CoOOH/CF, and CuO@CoOOH/CF were derived from CV curves obtained at different scan rates and found to be 2.73, 29.8, 41.9, and 86.4 mF cm^−2^, respectively (Figure 4b). This finding suggests the availability of more OER‐active sites in the CuO@CoOOH heterojunction electrocatalyst. The LSV curves were normalized to the ECSA values (Figure S11, Supporting Information), confirming that CuO@CoOOH/CF owns the highest intrinsic OER activity among the prepared samples (Figure S12, Supporting Information).

Electrochemical impedance spectroscopy (EIS) was recorded to study the charge transfer ability of the electrocatalysts.^[^ ^31^ ^]^ EIS was performed and fitted to an R(QR)(QR) equivalent circuit (Figure 4c, Table S3, Supporting Information), where *R*_s_ represents the solution resistance, *R*′ corresponds to the charge‐transfer resistance and *R*″ refers to the gas‐adsorption resistance.^[^ ^32^ ^]^ CuO@CoOOH/CF exhibits a smaller *R*_s_ (1.17 Ω) and *R*′ (0.14 Ω) as compared with the other samples, demonstrating its favorable charge transfer kinetics. The linear portions of the Tafel slopes were obtained from the Tafel equation (*η* = *b* log *j* + *a*, where *j* is the current density and *b* is the Tafel slope) to acquire further insights into the reaction kinetics of the OER process (Figure 4d and Table S4, Supporting Information). Compared with Cu(OH)_2_/CF (243.2 mV dec^−1^), CuO/CF (170.1 mV dec^−1^), and CoOOH/CF (104.2 mV dec^−1^), CuO@CoOOH/CF exhibits a much smaller Tafel slope (51.7 mV dec^−1^), which suggests enhanced reaction kinetics toward the OER. Multistep chronopotentiometry demonstrates that the CuO@CoOOH/CF features excellent mass transport properties and electrical conductivity during the OER process (Figure 4e). Besides catalytic activity, stability and durability are also important factors when assessing the practical application of an efficient electrocatalyst. The polarization curve of CuO@CoOOH/CF shows very slight degradation after 1000 cycles (inset, Figure 4f). Moreover, the potential can be maintained at ≈1.5 V (vs RHE) for the OER process at a constant current density of 20 mA cm^−2^ for a long period of 90 h.

The electrocatalytic performance of the as‐prepared catalysts for the hydrogen evolution reaction (HER) was also assessed in 1.0 m KOH (Figure S13, Supporting Information). CuO@CoOOH/CF shows high catalytic activity with a very low overpotential of 171 mV at a current density of −10 mA cm^−2^. Inspired by the excellent bifunctional electrocatalytic activity of the as‐prepared materials for OER and HER, CuO@CoOOH/CF was used as both the anode and cathode in 1.0 m KOH for overall water splitting (OWS). The CuO@CoOOH/CF || CuO@CoOOH/CF system bears good OWS activity with a low cell voltage of 1.633 V at a current density of 10 mA cm^–2^ (Figure S14, Supporting Information). Moreover, the electrolyzer shows negligible overpotential loss even after 100 h (Figure S15, Supporting Information), suggesting its good catalytic stability and durability. In addition, large‐scale CuO@CoOOH can be fabricated on CF (14 × 25 cm^2^) under laboratory conditions (Figure S16, Supporting Information), and the electrodes cut from this large‐scale sample have uniform electrocatalytic performance for OER (Figure S17, Supporting Information). This finding is an important criterion for estimating the industrial applicability of the prepared materials from laboratory‐scale to large‐scale production (Table S5, Supporting Information).

**Figure 5:**
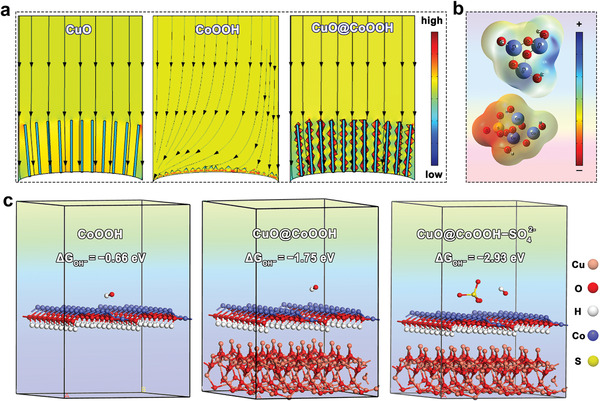
a) Current density distribution around the working electrode of CuO, CoOOH, and CuO@CoOOH. b) Electron cloud density distribution of CoOOH and CoOOH with surface adsorbed SO_4_^2−^. c) Optimized geometric structures of CoOOH, CuO@CoOOH heterojunction, and CuO@CoOOH heterojunction with surface adsorbed SO_4_^2−^.

To better understand the function of the formed heterojunction, current density distribution around the working electrode was simulated. As shown in **Figure** **5a**, when a certain potential was applied to the whole system, the current density distribution at the surface of the CuO@CoOOH electrode became much higher than that around the CoOOH electrode and CuO electrode. Thus, the heterojunction structure can accelerate the electron transfer at the interface.

Moreover, the nanoarray structure can guide the ions flow and weaken the large overpotential caused by the surface potential gradient,^[^ ^33^ ^]^ thereby enhancing the local electric field and accelerating the alkaline OER kinetics. Because OH^−^ adsorption is a critical step for the OER process,^[^ ^10^, ^34^ ^]^ electron cloud density distribution was evaluated to investigate the effect of surface adsorbed SO_4_^2−^ on the adsorption of OH^−^. As illustrated in Figure 5b, without SO_4_^2−^ adsorption, the charge distribution is relatively uniform according to the electron cloud density distribution of CoOOH. With SO_4_^2−^ adsorption, however, the negative charge centers are transferred onto SO_4_^2−^ and more positive charges are distributed around Co atom, thus promoting OH^−^ adsorption.

Generally, it has been proposed that OH^−^ was initially adsorbed on the surface of catalysts during the electrocatalytic OER in alkaline solution, and the initial adsorption of OH^−^ is crucial for the subsequent processes.^[^ ^10^, ^35^ ^]^ We also conducted density functional theory (DFT) calculations to confirm the quantitative adsorption‐promoting effects of the heterojunction structure and the surface adsorbed SO_4_^2−^ by using adsorption locator code (Figure 5c). When OH^−^ was adsorbed on the surface of CoOOH, the calculated adsorption energies on CoOOH and CuO@CoOOH were −0.66 and −1.75 eV, respectively. In the presence of SO_4_^2−^, CuO@CoOOH presented an even lower adsorption energy (i.e., −2.93 eV), allowing a facilitated OH^−^ adsorption on the surface of CoOOH. This result reveals that the p‐n heterojunction and surface‐adsorbed SO_4_^2−^ can help accelerate the electron transfer at the interface and boost the adsorption of OH^−^, consequently promoting the OER.

As the feature of the solid–liquid contact interface between catalysts and electrolyte plays an important role in the OER catalysis, a high‐speed digital camera system was used to identify the surface wettability of the as‐prepared electrocatalysts using a water droplet (100 µL) and monitor the releasing behaviors of produced bubbles at 1.43 V (vs RHE) (Figure S18, Supporting Information). It is found that CuO@CoOOH is more hydrophilic than CuO and CuO@CoS*_x_*, which contributes to the adsorption of reactants and diffusion of electrolytes.^[^ ^36^ ^]^ In addition, relatively larger O_2_ bubbles are adhering to the surface of the CuO and CuO@CoS*_x_*, which seriously hinder the access of water onto the electrode surface. As a sharp contrast, the surface of the CuO@CoOOH exhibits faster evolution and smaller O_2_ bubbles during the electrocatalytic reaction. Furthermore, the underwater gas‐bubble contact angle also suggests a weaker adhesion of bubbles on the CuO@CoOOH electrode. The desirable hydrophilicity of the electrocatalyst with the electrolyte and the enhanced surface aerophobicity of the gas bubbles also contribute to enhanced OER performance, in good agreement with the experimental and simulation results.

## 3 Conclusion

In summary, we have successfully developed a core–branch CuO@CoOOH p‐n heterojunction on CF (CuO@CoOOH/CF) as an efficient electrocatalyst for water splitting in alkaline solution. Various ex situ and in situ characterizations confirm that the heterojunction structure can promote the electron transfer and redistribution at the interface and the surface adsorbed sulfate ions released during the in situ anodic oxidation process can also contribute to the boosted electrocatalytic activity. The CuO@COOH/CF with surface‐adsorbed SO_4_^2−^ provides enhanced electrochemical OER activity (186 mV at 10 mA cm^−2^), improved charge transport capacity, fast reaction kinetics, and extraordinary long‐term stability. Given these properties, the as‐fabricated CuO@CoOOH p‐n heterojunction material is superior to most of the reported Co‐based OER catalysts in alkaline media. We believe this work introduces a favorable pathway for the design and synthesis of efficient non‐noble metal‐based p‐n heterojunction electrocatalysts with well‐defined structures and controllable compositions under laboratory conditions.

## Conflict of Interest

The authors declare no conflict of interest.

## Supporting information

Supporting Information

## Data Availability

Research data are not shared.
