# Supplementary material for: Improved Interface Charge Transfer and Redistribution in CuO‐CoOOH p‐n Heterojunction Nanoarray Electrocatalyst for Enhanced Oxygen Evolution Reaction
Source: Adv Sci (Weinh). 2021 Oct 12;8(22):2103314. doi: 10.1002/advs.202103314 (PMC8596130; doi:10.1002/advs.202103314)
Supplement: Supplementary file 1 — Supporting Information [file ADVS-8-2103314-s001.pdf]

© 2021 Wiley-VCH GmbH

ADVANCED SCIENCE
Open Access

Supporting Information

for *Adv. Sci.,* DOI: 10.1002/advs.202103314

Improved Interface Charge Transfer and Redistribution in CuO-CoOOH p-n Heterojunction Nanoarray Electrocatalyst for Enhanced Oxygen Evolution Reaction

Jing Hu[1], Adel Al-Salihy[1], Jing Wang[1], Xue Li[1], Yanfei Fu[1], Zhonghua Li[1], Xijiang Han[1], Bo Song[2] and Ping Xu[1,]*

Electronic Supplementary Information

Improved Interface Charge Transfer and Redistribution in CuO-CoOOH p-n Heterojunction Nanoarray Electrocatalyst for Enhanced Oxygen Evolution Reaction

Jing Hu[1], Adel Al-Salihy[1], Jing Wang[1], Xue Li[1], Yanfei Fu[1], Zhonghua Li[1], Xijiang Han[1], Bo Song[2] and Ping Xu[1,]*

[1] MIIT Key Laboratory of Critical Materials Technology for New Energy Conversion and Storage, School of Chemistry and Chemical Engineering, Harbin Institute of Technology, Harbin 150001, PR China.

[2] National Key Laboratory of Science and Technology on Advanced Composites in Special Environments, Harbin Institute of Technology, Harbin 150001, PR China.

*Corresponding author. Email: pxu@hit.edu.cn

Theoretical Simulation and Calculation.

Density functional theory (DFT) calculations:

We used the generalized gradient approximation (GGA) with the Perdew-Burke-Ernzerhof (PBE) exchange correlation function and a 320 eV cutoff energy for the plane-wave basis set to calculate the adsorption energy through Materials Studio software and Dmol3 module using the Energy cutoff option on the Electronic tab of the Calculation dialog.[1] The interaction between valence electrons and the ionic core is described by On-The-Fly-Generated (OTFG) ultra-soft pseudopotential which is found in the CASTEP code. The theoretical models were employed to simulate the materials of CuO@CoOOH, CuO and CoOOH using Crystal Maker. The similar geometries have been used to make the molecule most stable for generating an optimal (lowest energy) structure from an arbitrary starting state.

Current density distribution simulations:

The COMSOL 5.4 software and the one-dimensional linear electrical analysis(elan) model have been used to simulated the current density distribution. First, the square regions with DC voltage differences applied that represent the electrolytic cells have been constructed (1.2 μm*1.6μm) to simulate the filling of the electrolyte. The arc surface below represents the electrode surface, where the sharp triangle-like shape on the surface represents CoOOH, and arrays that covered by

the above shape represent the CoOOH supported by CuO nanoarrays, the bare arrays represent CuO nanoarrays.

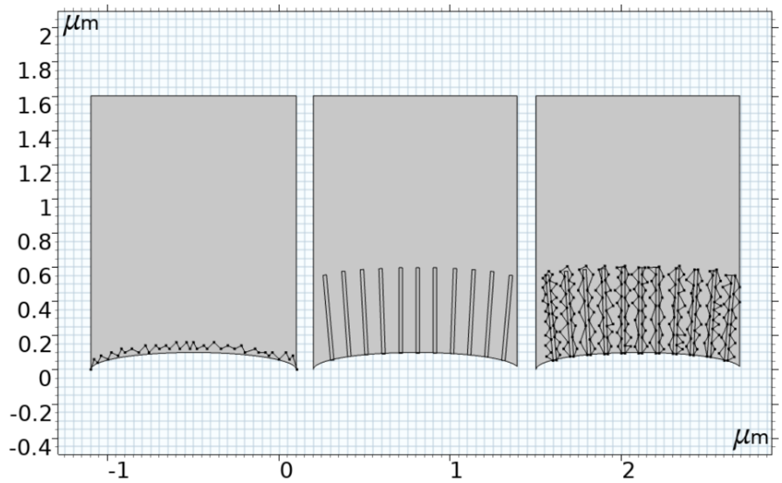

Electric Currents (EC) module and Stationary study has been used to improve the simulation precision. The appropriate simulation precision has been set through using the Finer Mesh.

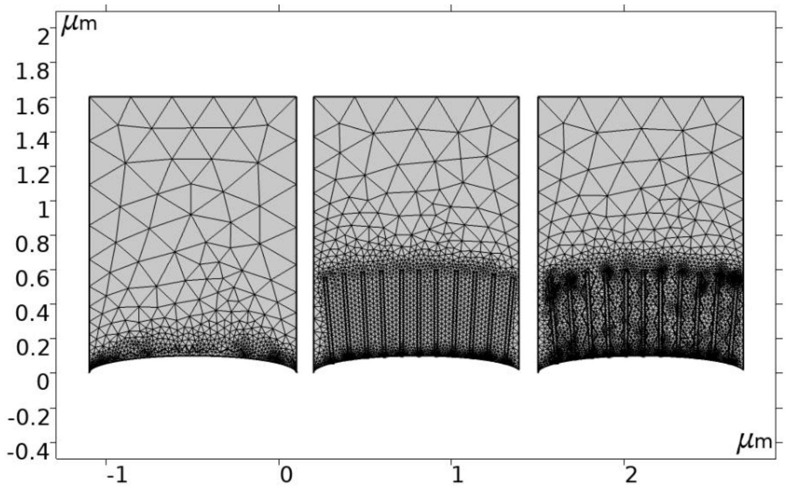

In planar 2D, the Electric Potential interface assumes that the model has a symmetry where the electric current varies only in the *x* and *y* directions and is constant in the *z* direction. This implies that the electric field, E, is tangential to the *xy*-plane. In 2D axi-symmetry, the Electric Potential interface considers the situation where the fields and geometry are axially symmetric. In this case, the electric current is constant in the φ direction, which implies that the electric field is tangential to the *rz*-plane.

The Potential Conservation was also added to the continuity equation for the electrical current density and provides an interface for defining the electric conductivity as well as the constitutive relation and the relative permittivity for the displacement current.

Parameters.

Conductivity of CuO nanoarrays: 10^{-4} S/ cm^{-1};[2]

Conductivity of CoOOH: 12.8 S cm^{-1}.[3]

Experimental Section

Chemicals and Reagents.

Copper foam (CF) (thickness: 2 mm, bulk density: 0.58 g/cm^3, pore size: 0.1 mm, number of pores per inch: 90) was purchased from Kunshan Longshengbao Electronic Materials Co., Ltd. Ammonium persulfate [APS, $(NH_4)_2S_2O_8$, ≥98.0%], cobalt (II) nitrate hexahydrate ($Co(NO_3)_2{\cdot}6H_2O$, 99%), potassium hydroxide (KOH, 95%), and sodium hydroxide (NaOH, 97%) were purchased from Aladdin Reagent. Thioacetanilide [TAA, C_8H_9NS, ≥98.0%] was purchased from Tianjin Guangfu Fine Chemical Research Institute. Commercial Iridium dioxide (IrO_2) and commercial Pt/C (20% Pt on Vulcan XC-72) were purchased from Alfa Aesar. Ethyl alcohol and acetone were purchased from Tian in Fuyu Fine Chemical Co., Ltdj. Deionized water (from MilliQ system) with a resistivity of 18 MΩ·cm at 25 ^oC was used in all experiments. All chemicals and reagents were used as received without any further purification.

Pre-treatment of Cu foam

A piece of CF was cut into an area of 1 × 2 cm^2, and then the CF was washed with acetone, HCl (3 M), ethanol and deionized water, respectively, for 10 min with the assistance of ultrasonication for several times to clean the CF's surface for further use before dried in a vacuum oven.

Synthesis of $Cu(OH)_2$/CF and CuO/CF.

The $Cu(OH)_2$ nanoarrays were prepared by a typical controlled in-situ oxidative etching method at 25 ^oC.[4] Briefly, A certain amount of NaOH (80 mmol) was added into 30 mL deionized water under stirring to make a transparent solution, in which APS (3 mmol) was added. Then the cleaned CF was immersed into the as-prepared solution at 25 ^oC ($S_2O_8^{2-} + Cu \rightarrow 2SO_4^{2-} + Cu^{2+}$). After a given reaction time, the sample was taken out of the solution, washed with deionized water three times and alcohol twice, and dried in air. For the preparation of CuO nanoarrays, the as-prepared $Cu(OH)_2$ nanoarrays were dried and calcined at 180 °C for 3h. Different processing times have been tested to get the best experimental process parameters (Figure S19-S22 and Table S6, Supporting Information).

Synthesis of CuO@CoOOH/CF.

The CuO@CoOOH nanocomposites were prepared by a chemical bath deposition technique and subsequent in-situ anodic oxidation process. Briefly, $Co(NO_3)_2{\cdot}6H_2O$ and TAA in a molar ratio of 1:2 were added into 50 mL ethanol under stirring to make a transparent solution. The cleaned $Cu(OH)_2$/CF was then immersed in the as-prepared solution. After a given reaction time at 72 °C, the prepared sample of CuO@CoS_x/CF was taken out from the solution, and then washed

three times in deionized water and twice in alcohol, and dried in air. The CuO@CoOOH was then achieved by an in-situ anodic oxidation strategy using cyclic voltammetry test measured in the potential range at a scan rate of 50 mV s^{-1} in 1.0 M KOH solution for 50 cycles. The voltage window for cycling is 1.124 to 1.924 V *vs*. RHE. The processing time was further optimized in terms of the OER performance (Figure S23-S26 and Table S7, Supporting Information). Additionally, the sample with a larger size (14 × 25 cm^2) was prepared using the identical method under laboratory conditions.

Synthesis of CoOOH/CF

CoOOH/CF was prepared using a similar procedure to that for the synthesis of CuO@CoOOH/CF. The only difference between them lies in the use of a CF instead of a $Cu(OH)_2$/CF.

Characterizations

Powder X-ray diffraction (XRD) patterns were obtained on a Rigaku D/MAXRC X-ray diffractometer using a Cu Kα radiation source (40.0 kV, 40.0 mA). The surface morphologies of the samples were characterized by a field-emission scanning electron microscope (FESEM; Zeiss Supra 55) with a voltage of 10.0 kV and a working distance of 8.7 mm. All SEM images were recorded with an ET secondary electron detector. Transmission electron microscopy (TEM) was performed on a Tecnai F20 instrument equipped with a field-emission gun operated at an accelerating voltage of 200.0 kV. Raman spectra were collected with a confocal Raman spectroscopic system (Renishaw, InVia) using a 532 nm laser with a TE air-cooled 576 × 400 CCD array as the excitation source. X-ray photoelectron spectroscopy (XPS) was carried out on an ESCA Lab MKII X-ray photoelectron spectrometer using Al Kα radiation as an excitation source (hυ = 1486.6 eV). A surface area analyzer (AUTOSORB-1, Quantachrome Instruments) was used to measure N_2 physisorption isotherms at 77 K. Specific surface areas were estimated by means of the Brunauer–Emmett–Teller method method, and pore-size distributions were calculated from the adsorption branch of the isotherm by means of the Barrett–Joyner–Halenda method.

Electrode Preparation and Electrochemical Measurements

All electrochemical measurements were performed with a standard three-electrode system on a CHI660E (electrochemical workstation CHI 660E), in which the as-prepared samples on Cu foam (1×1 cm^2) ($Cu(OH)_2$/CF, CoS_x/CF, and CuO@CoS_x/CF) were used directly as the working electrode. A Hg/HgO electrode and a graphite rod (Alfa Aesar, 99.999%) were used as the reference and counter electrode, respectively. Prior to the electrochemical measurements, the Hg/HgO electrode was experimentally calibrated and referred to the reversible hydrogen

electrode (RHE) in 1.0 M KOH solution according to previous report (Figure S27, Supporting Information).[5] All electrochemical measurements were conducted in an electrolyte of 1.0 M KOH solution, and the results were *i*R-corrected. Linear sweep voltammetry (LSV) was performed at a slow scan rate of 1 mV s^{-1} to eliminate the generation of a capacitive current during the electrocatalytic reaction. Electrochemical impedance spectroscopy (EIS) wasconducted at an amplitude of 5 mV over the frequency range of 10^6 – 0.1 Hz at an AC potential of –1.1 V for the HER and 1.45 V for the OER. The series resistance obtained from the EIS measurements was used to correct the ohmic drop during the test and achieve a better comparison the catalytic activity of the different catalysts. *i*R compensation was performed by applying the following equation, E(*i*R) = E(Hg/HgO) − *i*R. The Mott-Schottky plots were obtained in the above mentioned three-electrode system at an amplitude of 10 mV and a fixed frequency of 1 kHz.

Estimation of electrochemical active surface area (ECSA).

The capacitive currents were performed within a potential range where no faradic reactions occurred. Cyclic voltammograms at different scan rates (5, 10, 15, 20, 25, 30, 35, 40, 45 and 50 mV s^{-1}) were collected in the range of 0.924-1.024 V vs. RHE to estimate the double-layer capacitance (C_{dl}). By plotting the differences in current density variation ($\Delta j = j_a - j_c$) at the potential of 0.1 V vs. RHE against scan rate, a linear trend of the electrochemical double-layer capacitance (C_{dl}) was fitted, which was proportional to the electrochemical active surface area (ECSA). As the specific capacitance for a flat surface is generally computered to be in the range of 20~60 μF cm^{-2}, therefore 40 μF cm^{-2} was used in the following calculations of the ECSA as generally reported in articles.[6]

The ECSA is calculated according to the following formula:

$$\mathrm{ECSA} = \frac{C_{dl}}{40\ \mu\mathrm{F}\cdot\mathrm{cm}^{-2}\ \mathrm{per}\ \mathrm{cm}^{2}}$$

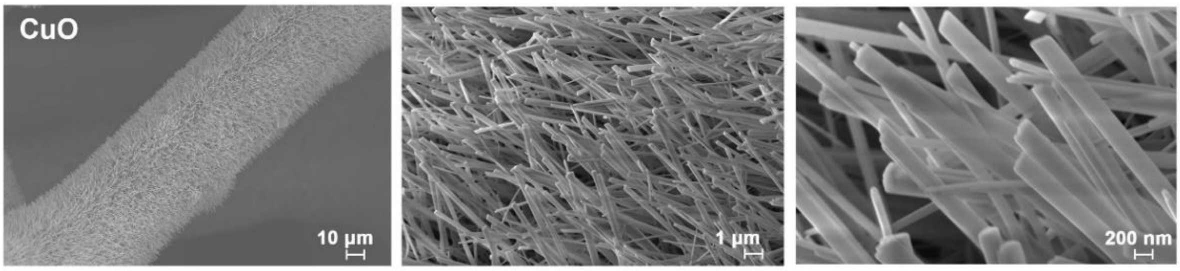

Figure S1. SEM images of CuO on Cu foam.

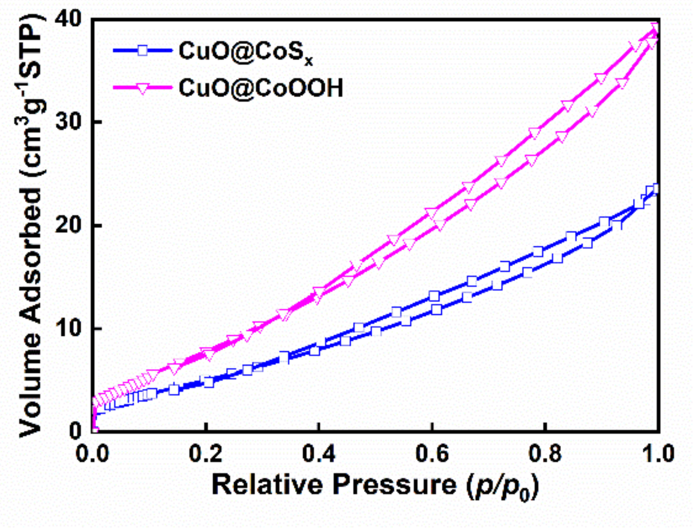

Figure S2. Nitrogen adsorption–desorption isotherms of the prepared samples.

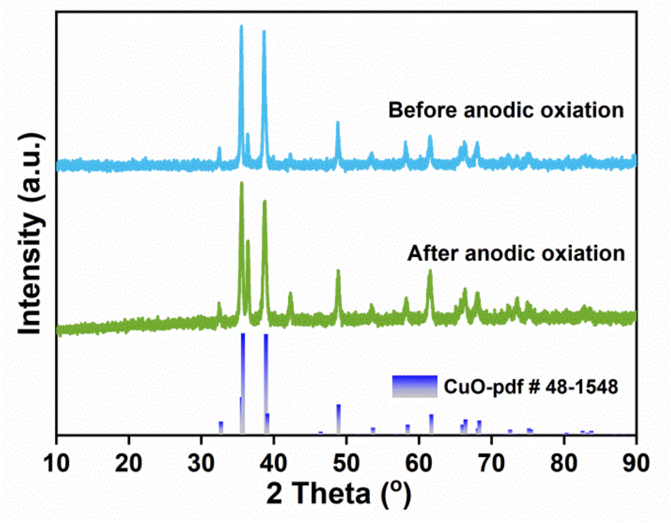

Figure S3. XRD patterns of the prepared samples that scraped down from the Cu foam.

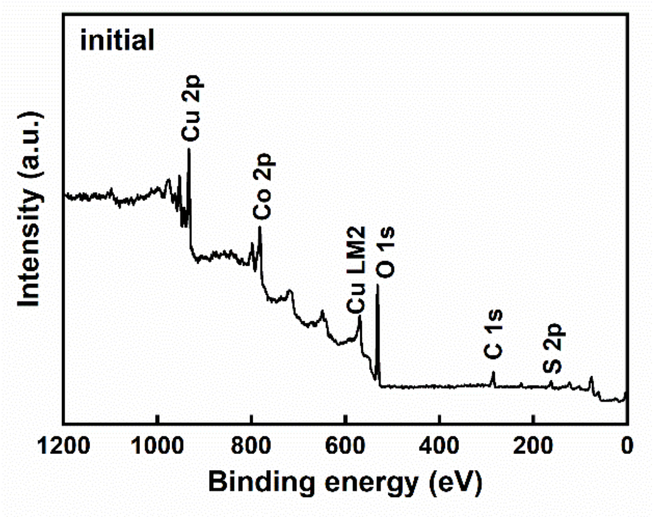

Figure S4. Survey XPS spectrum of CuO@CoS$_x$/CF.

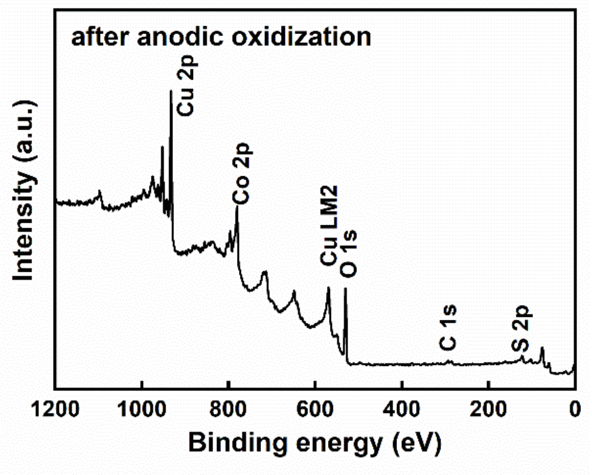

Figure S5. Survey XPS spectrum of CuO@CoOOH/CF with SO_4^{2-} adsorbed on the surface.

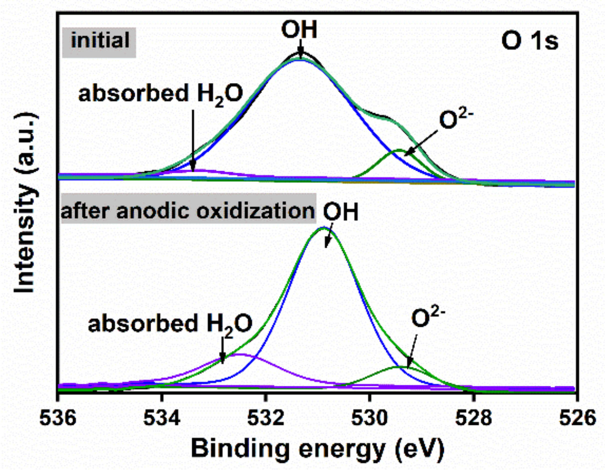

Figure S6. O 1s XPS spectra of CuO@CoS$_x$/CF before and after the anodic oxidization process.

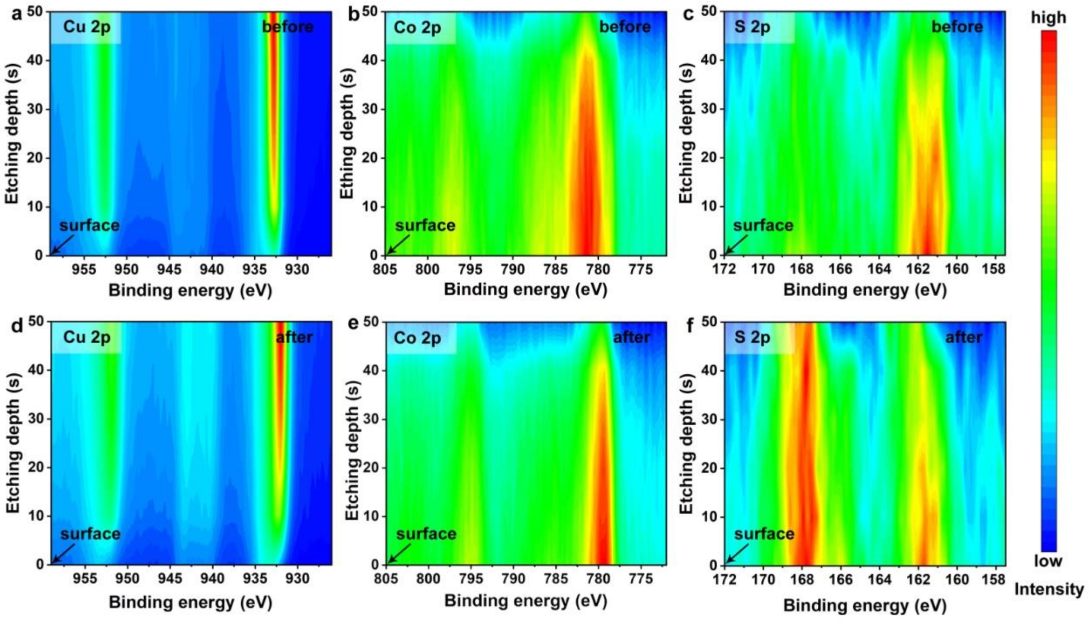

Figure S7. (a) The heat map of the XPS analysis of Cu, Co, and S with different etching depth before (a, b, c) and after (d, e, f) the anodic oxidation process.

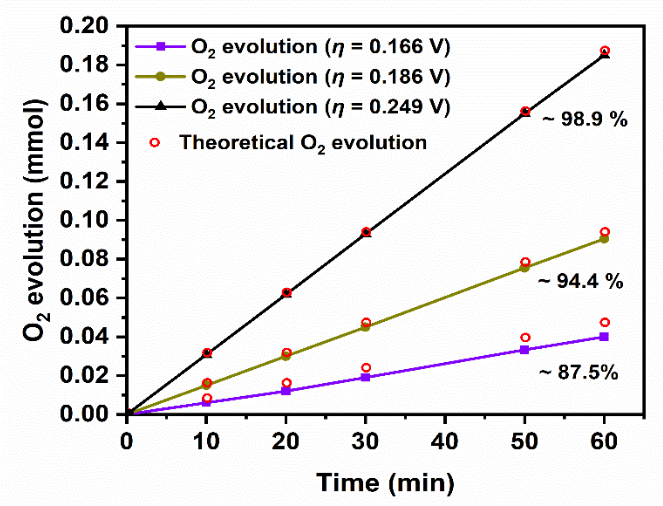

Figure S8. Faradaic efficiency of oxygen production over CuO@CoOOH at overpotential of 0.166 V, 0.186 V and 0.249 V, respectively.

According to Figure S8, the Faradaic efficiency of CuO@CoOOH was 87.5%, 94.4%, and 98.9%, respectively, at the overpotential of 0.166 V, 0.186 V, and 0.249, which shows that besides the OER reaction, the slow current increase stage may include electric double-layer charging current (ion adsorption and migration).

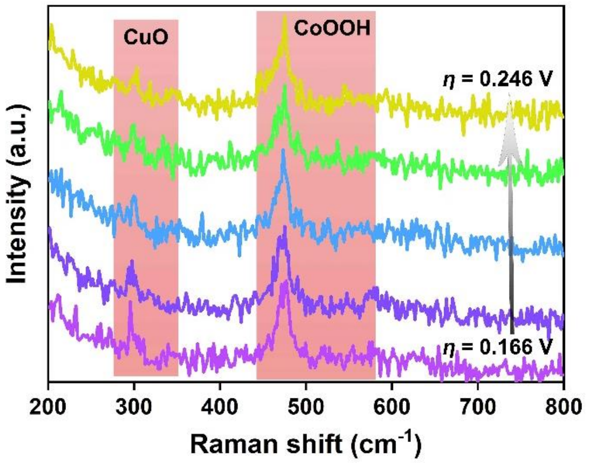

Figure S9. In-situ Raman spectra of CuO@CoOOH from the overpotential of 0.166 V to 0.246 V during the LSV test.

In order to verify whether the substrate material has been oxidized under low current, in-situ Raman spectra and XRD patterns were achieved. As can be seen from the raman spectra, when testing the LSV from the low potential to high potential with a scan rate of 1 mV s^{-1} (in the overpotential range from 0.166 V to 0.246 V), only peaks of CuO and CoOOH were achieved, and no significant changes was observed in the intensity of the peaks.

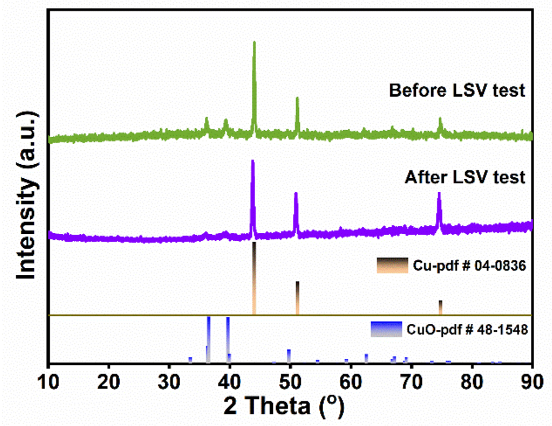

Figure S10. XRD patterns of CuO@CoOOH before and after the LSV (from the overpotential of 0.166 V to 0.249 V).

In addition, XRD patterns before and after the LSV test (from the overpotential of 0.166 V to 0.249 V) were also achieved. As can be seen, there also no changes been detected during the LSV test from the overpotential of 0.166 V to 0.249 V. In view of this, it is said that the slow current increase stage could simple be from the electric double-layer charging current (ion adsorption and migration). Therefore, the overpotential at current density of 20 mA cm^{-2} had been chosen to more accurately express and compare the performance of the catalysts.

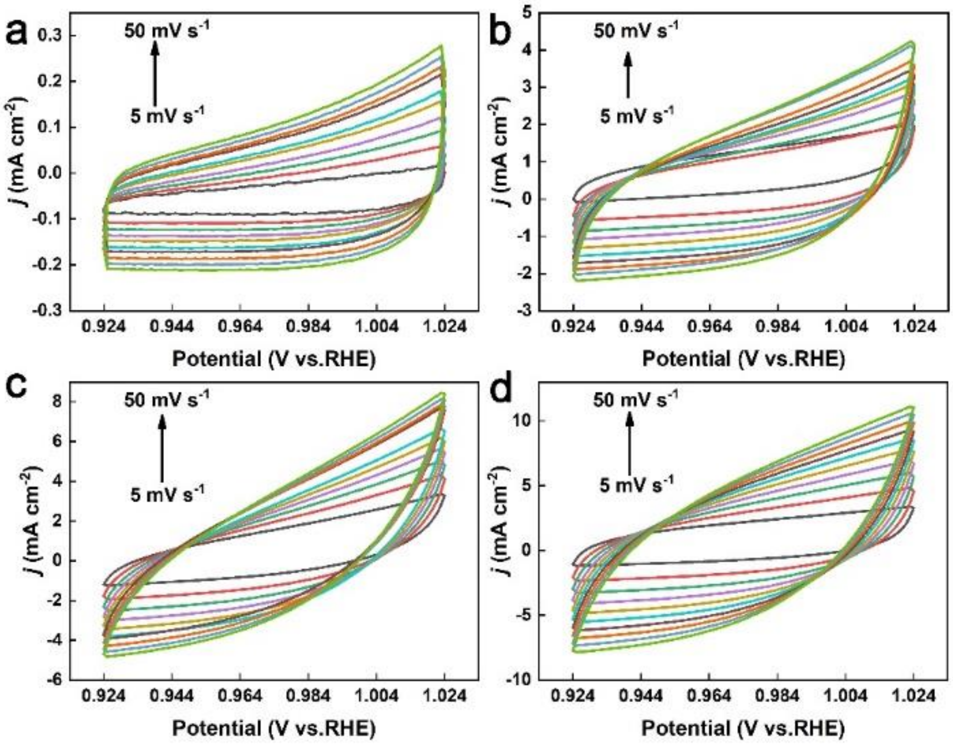

Figure S11. CV curves of $Cu(OH)_2$/CF (a), CuO/CF (b), CoOOH/CF (c), and CuO@CoOOH/CF (d) at various scan rates (5~50 mV s^{-1}) in the potential range of 0.924~1.024 V vs. RHE, which were used to estimate the double-layer capacitance (C_{dl}).

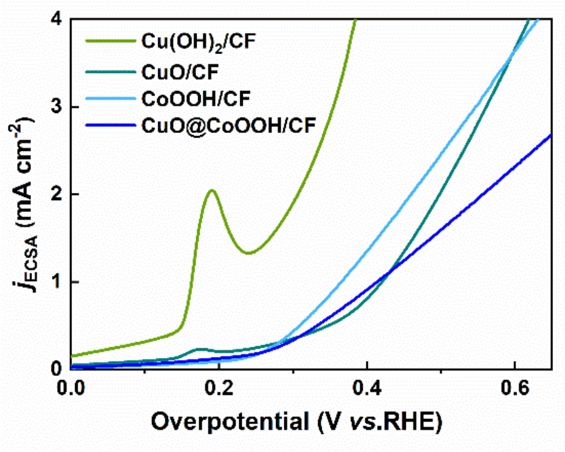

Figure S12. ECSA-normalized OER polarization curves of the prepared samples.

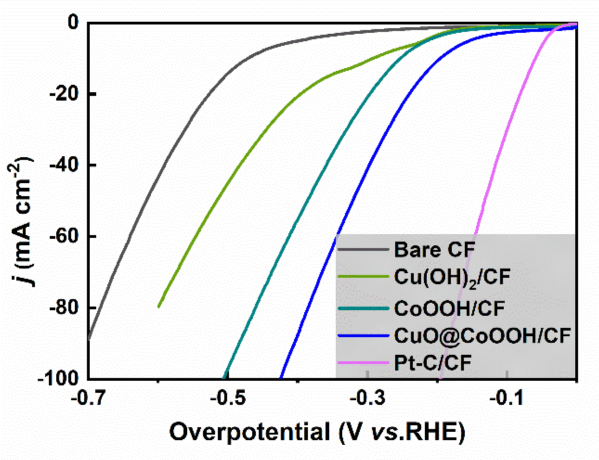

Figure S13. HER polarization curves of the prepared samples.

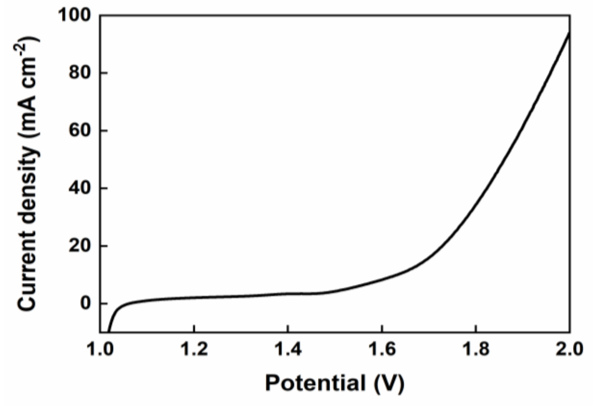

Figure S14. Overall Water Splitting activities of CuO@CoOOH/CF.

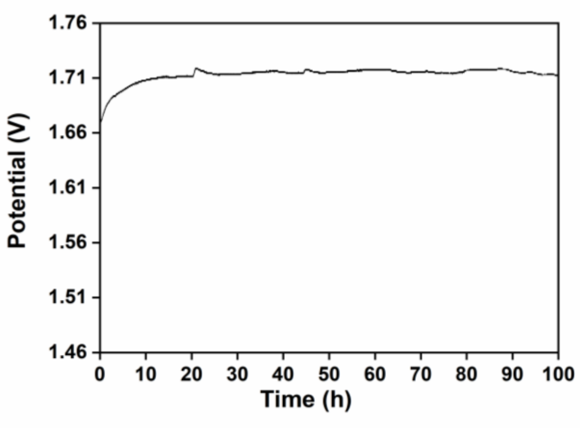

Figure S15. Long-term stability of the CuO@CoOOH/CF||CuO@CoOOH/CF electrode in an alkaline electrolyzer under a constant current density of 10 mA cm^{-2}, measured for 100 h.

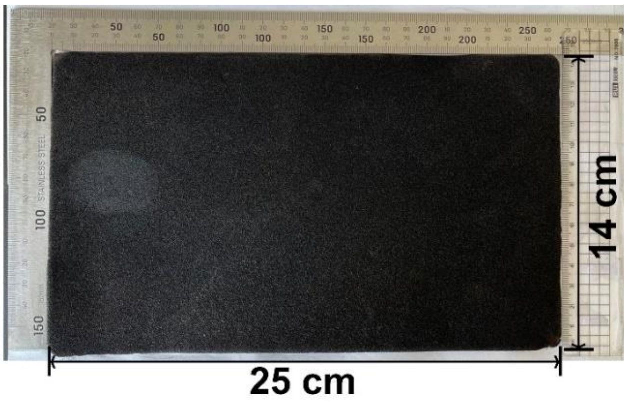

Figure S16. Digital image of the CuO@CoOOH/CF with a larger area of 14 × 25 cm^2.

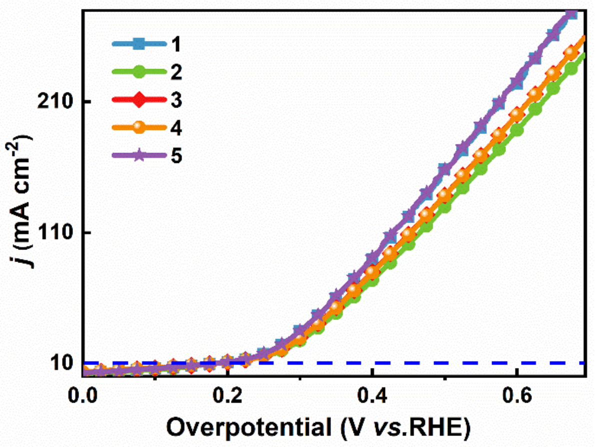

Figure S17. LSV curves measured from five different parts cut from the same large-scale sample of the CuO@C@CoOOH nanocomposites for OER catalysis in 1 M KOH solution.

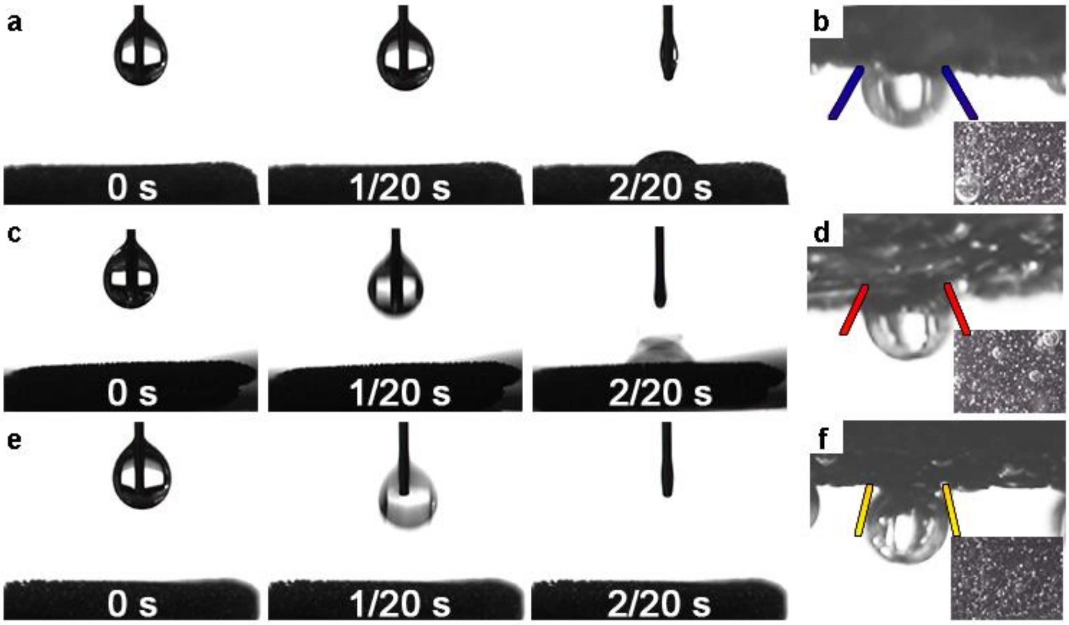

Figure S18. Contact angle photograph of the droplet experiment of (a) CuO/CF, (c) CuO@CoS_x/CF, (e) CuO@CoOOH/CF. The images of bubbles detaching and bubble contact angles on different electrodes of (b) CuO/CF, (d) CuO@CoS_x/CF, (f) CuO@CoOOH/CF.

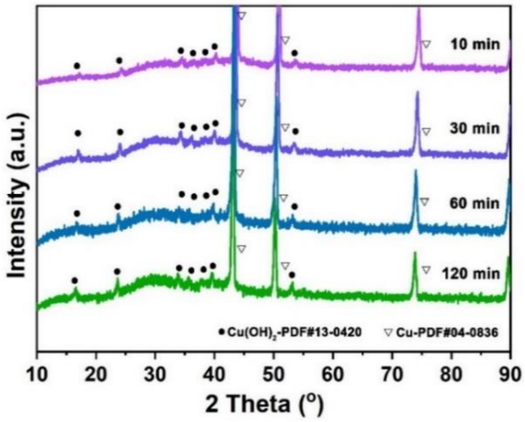

Figure S19. XRD patterns of samples of $Cu(OH)_2$ with different reaction time.

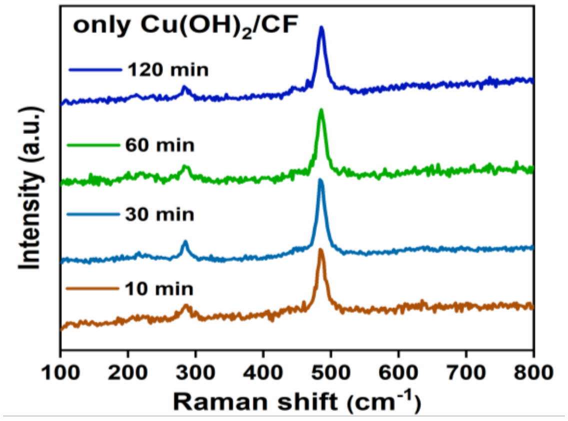

Figure S20. Raman spectra of the samples of $Cu(OH)_2$ with different reaction time.

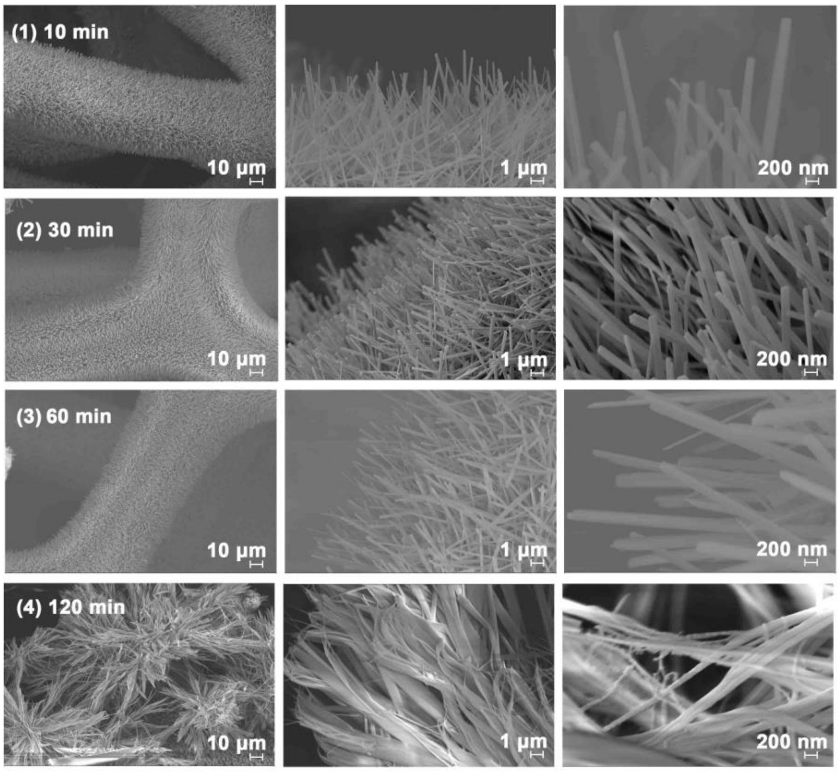

Figure S21. SEM images of the samples of $Cu(OH)_2$ with different reaction time.

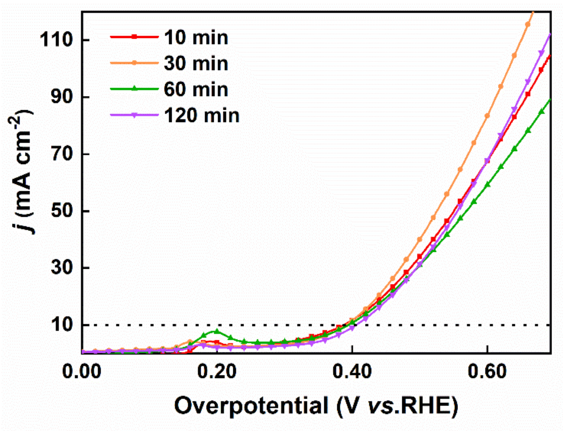

Figure S22. Linear sweep voltammetry (LSV) curves of the samples of $Cu(OH)_2$ after *iR* correction with different reaction time for OER in 1.0 M KOH.

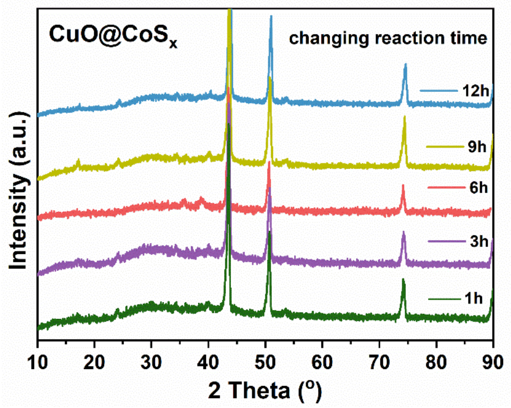

Figure S23. XRD patterns of samples of CuO@CoS$_x$ with different reaction time.

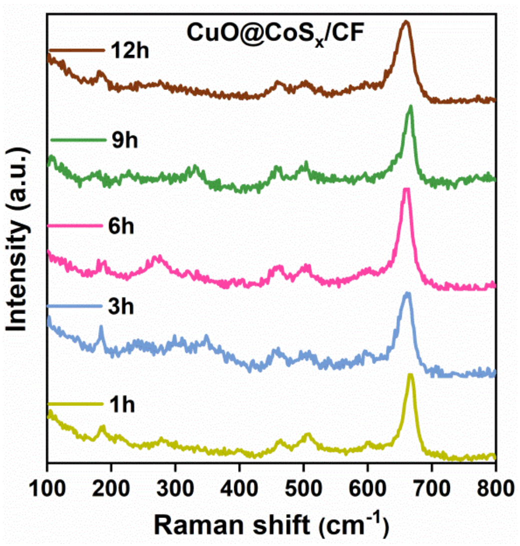

Figure S24. Raman spectra of samples of CuO@CoS$_x$ with different reaction time.

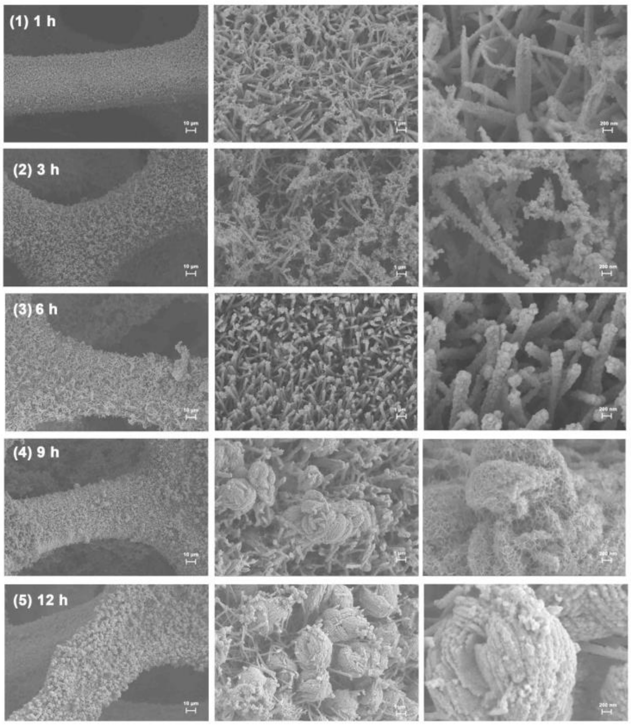

Figure S25. SEM images of samples of CuO@CoS$_x$ with different reaction time.

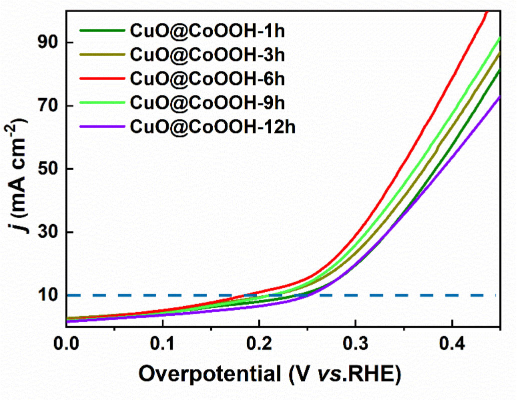

Figure S26. Linear sweep voltammetry (LSV) curves of samples of CuO@CoOOH after *iR* correction with different reaction time for OER in 1.0 M KOH.

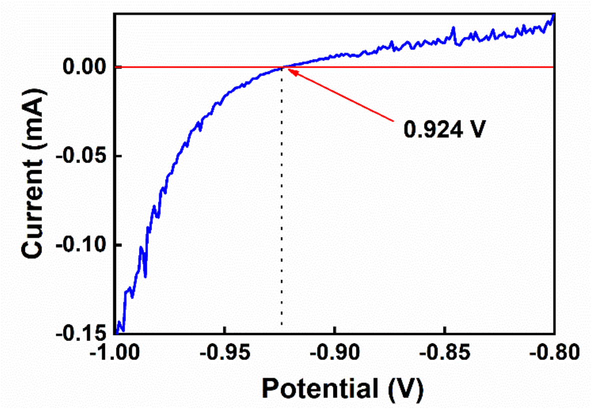

Figure S27. Electrode calibration LSV curve of Hg/HgO in 1.0 M KOH (scan rate: 1 mV s^{-1}) at temperature of 20 °C.

Table S1. The textural parameters of CuO@CoS_x and CuO@CoOOH determined by N_2 adsorption–desorption isotherms.

Samples	BET surface Area (m^2/g)	Pore Diameter (nm)	Pore Volume (cm^3/g)
CuO@CoS_x	21.384	2.761	0.0456
CuO@CoOOH	36.355	1.7318	0.0088

Table S2. Comparison of the OER performance for CuO@CoOOH/CF with other related electrocatalysts.

Numbers	Materials	Overpotential (at 10 mA cm^{-2})	Tafel slope (mV dec^{-1})	References
1	CuO@CoOOH/CF	186 mV	51.7	This work
2	Fe doped CoOOH Nanosheet Arrays	266	30	Reference[7]
3	Ni-CoOOH	~280	36	Reference[8]
4	Ultrathin CoOOH nanosheets	253	87	Reference[9]
5	Mn-doped CoOOH nanosheets	255	33	Reference[10]

6	bulk CoOOH	370	69	Reference[11]
7	Ag-Doped CoOOH nanosheet	256	64.6	Reference[12]
8	Mo_1-CoOOH@CP	274	66	Reference[13]
9	FeCoOOH/NF	211	33	Reference[14]
10	Plasma-engraved CoOOH	262	42	Reference[15]
11	CoOOH-graphene	248	32	Reference[16]
12	The CoOOH nanodisks	245	53.7	Reference[17]
13	FeCoOOH-nanosheets	230	54	Reference[18]
14	CoOOH hollow nanospheres	275	56.1	Reference[19]
15	$Co(OH)_2$/CoOOH Heterostructure	395	63	Reference[20]
16	Au-Decorated CoOOH nanoplates	320	57.2	Reference[21]
17	CoOOH HNSs	305	63	Reference[22]
18	Ni-Fe-OH@Ni_3S_2/NF	165	93	Reference[23]
19	Ni_3FeN-NPs	280	46	Reference[24]
20	S-$NiFe_2O_4$/NF	267	36.7	Reference[25]
21	$Ni_{0.6}Co_{1.4}P$	300	80	Reference[26]
22	NiO/$NiFe_2O_4$	302	42	Reference[27]
23	RuO_2/Co_3O_4	305	69	Reference[28]
24	Ni_3FeN-NPs	280	46	Reference[29]
25	$MnGa_4$/NF	291	98	Reference[30]
26	$Sr_3FeCoO_{7-\delta}$	343 ± 4.4 mV	63	Reference[31]
27	$CaFeO_3$	390	47	Reference[32]
28	NiFe-UMNs	260	30	Reference[33]
29	NiFe-LDH-CNTs	250	31	Reference[34]
30	NiCo-UMOFNs/Cu foam	189	42	Reference[35]
31	Co-MoS_2/BCCF-21	260	85	Reference[36]
32	MoS_2/Ni_3S_2	218	88	Reference[37]
33	Co/Ni-1T-MoS_2	235	45.7	Reference[38]
34	Ni-Fe oxide/Ni foam	195	28	Reference[39]
35	S-incorporated $Ni_{6/7}Fe_{1/7}$-	190	24	Reference[40]

	OH/CNT/glassy carbon

Table S3. Impedance parameter values derived from the fitting to the equivalent circuit for the impedance spectra recorded in 1.0 M KOH solution.

Smaples	R_s[Ω]	R' [Ω]	Q'[Ss^{-n}]	n1	R'' [Ω]	Q''[Ss^{-n}]	n2
$Cu(OH)_2$/CF	1.33	0.51	1.3*10^{-2}	0.86	217	1.7*10^{-2}	0.71
CuO /CF	1.38	0.33	3.2*10^{-2}	0.84	279	3.8*10^{-2}	0.78
CoOOH/CF	1.23	0.78	6.1*10^{-2}	0.87	822	7.1*10^{-2}	0.81
CuO@CoOOH/CF	1.17	0.14	5.2*10^{-1}	0.93	233	7.2*10^{-2}	0.89

Table S4. The Tafel slope and the exchange current density of the prepared samples.

Smaples	Tafel (mV dec^{-1})	j_0 (mA cm^{-2})
$Cu(OH)_2$/CF	243.2	0.07*10^{-2}
CuO /CF	170.1	0.13*10^{-2}
CoOOH/CF	104.2	0.4*10^{-2}
CuO@CoOOH/CF	51.7	4.5*10^{-2}

Table S5. Electrocatalysts that prepared with large sizes.

Numbers	Materials	Size (cm^2)	η /mV	References
1	Ag_3PO_4 (OER)	4.5 × 4.5	302(10 mA cm^{-2})	Reference[41]
2	Ni_2P/NF (OER)	10 × 10	300 (100 mA cm^{-2})	Reference[42]
3	NiO/Ni (OER)	10 × 10	294 (10 mA cm^{-2})	Reference[43]
4	γ-FeOOH/NF (OER)	100 × 20	286 (10 mA cm^{-2})	Reference[44]
5	Co/N@CNTs@CNMF-800 (OER)	200 × 100	310 (10 mA cm^{-2})	Reference[45]
6	FeNiW-LDH/Fe foam (OER)	16 × 23	202 (10 mA cm^{-2})	Reference[46]
7	CuO@CoOOH/CF (OER)	14 × 25	186 (10 mAcm^{-2})	This work

Table S6. Overpotential of the samples with different reaction time for OER in 1.0 M KOH.

Numbers	Samples	Overpotential (at 10 mA cm^{-2})
1	$Cu(OH)_2$/CF-10 min	391 mV
2	$Cu(OH)_2$/CF-30 min	376 mV

3	$Cu(OH)_2$/CF-60 min	395 mV
4	$Cu(OH)_2$/CF-120 min	406 mV

Table S7. Overpotential of samples of CuO@CoOOH with different reaction time for OER in 1.0 M KOH.

Numbers	Samples	Overpotential (at 10 mA cm^{-2})
1	CuO@CoOOH/CF(1 h)	240 mV
2	CuO@CoOOH/CF(3 h)	212 mV
3	CuO@CoOOH/CF(6 h)	186 mV
4	CuO@CoOOH/CF(9 h)	211 mV
5	CuO@CoOOH/CF(12 h)	251 mV

References

[1] K. He, T. Tadesse Tsega, X. Liu, J. Zai, X.-H. Li, X. Liu, W. Li, N. Ali, X. Qian, *Angew. Chem. Int. Ed.* **2019**, *58*, 11903.
[2] A. A. M. Sakib, S. M. Masum, J. Hoinkis, R. Islam, M. A. I. Molla, *J. Compos. Sci.* **2019**, *3*, 91.
[3] B. Guo, T. Li, H. Hu, *J. Appl. Electrochem.* **2016**, *46*, 403.
[4] X. Wen, W. Zhang, S. Yang, *Langmuir* **2003**, *19*, 5898.
[5] J. Hu, S. Li, Y. Li, J. Wang, Y. Du, Z. Li, X. Han, J. Sun, P. Xu, *J. Mater. Chem. A* **2020**, *8*, 23323.
[6] a) S.-Q. Liu, H.-R. Wen, G. Ying, Y.-W. Zhu, X.-Z. Fu, R. Sun, C.-P. Wong, *Nano Energy* **2018**, *44*, 7; b) Y. Yang, W. Zhang, Y. Xiao, Z. Shi, X. Cao, Y. Tang, Q. Gao, *Appl. Catal. B: Environ.* **2019**, *242*, 132.
[7] S.-H. Ye, Z.-X. Shi, J.-X. Feng, Y.-X. Tong, G.-R. Li, *Angew. Chem. Int. Ed.* **2018**, *57*, 2672.
[8] Z. Chen, C. X. Kronawitter, Y.-W. Yeh, X. Yang, P. Zhao, N. Yao, B. E. Koel, *J. Mater. Chem. A* **2017**, *5*, 842.
[9] J. Zhou, Y. Wang, X. Su, S. Gu, R. Liu, Y. Huang, S. Yan, J. Li, S. Zhang, *Energy Environ. Sci.* **2019**, *12*, 739.
[10] Y. Huang, X. Zhao, F. Tang, X. Zheng, W. Cheng, W. Che, F. Hu, Y. Jiang, Q. Liu, S. Wei, *J. Mater. Chem. A* **2018**, *6*, 3202.
[11] S. Song, H. Bao, X. Lin, X.-L. Du, J. Zhou, L. Zhang, N. Chen, J. Hu, J.-Q. Wang, *J. Energy Chem.* **2020**, *42*, 5.
[12] C. Lee, K. Shin, C. Jung, P.-P. Choi, G. Henkelman, H. M. Lee, *ACS Catal.* **2020**, *10*, 562.
[13] Y. Zhang, H. Guo, P. Yuan, K. Pang, B. Cao, X. Wu, L. Zheng, R. Song, *J. Power Sources* **2019**, *442*, 227252.
[14] T.-T. H. Nguyen, J. Lee, J. Bae, B. Lim, *Chem. Eur. J.* **2018**, *24*, 4724.
[15] J. Wang, J. Liu, B. Zhang, H. Wan, Z. Li, X. Ji, K. Xu, C. Chen, D. Zha, L. Miao, J. Jiang, *Nano Energy* **2017**, *42*, 98.
[16] F. Tang, W. Cheng, Y. Huang, H. Su, T. Yao, Q. Liu, J. Liu, F. Hu, Y. Jiang, Z. Sun, S. Wei, *ACS Appl. Mater. Interfaces* **2017**, *9*, 26867.
[17] J. Du, C. Li, X. Wang, T. G. J. Jones, H.-P. Liang, *Electrochim. Acta* **2019**, *303*, 231.
[18] S. Ibraheem, S. Chen, J. Li, Q. Wang, Z. Wei, *J. Mater. Chem. A* **2019**, *7*, 9497.
[19] H. Wang, E.-m. Feng, Y.-m. Liu, C.-y. Zhang, *J. Mater. Chem. A* **2019**, *7*, 7777.
[20] L. Liu, Y. Ou, D. Gao, L. Yang, H. Dong, P. Xiao, Y. Zhang, *J. Power Sources* **2018**, *396*, 395.
[21] S. Feng, L. Yang, Z. Zhang, Q. Li, D. Xu, *ACS Appl. Energy Mater.* **2020**, *3*, 943.
[22] Q.-L. Hong, Q.-G. Zhai, X.-L. Liang, Y. Yang, F.-M. Li, Y.-C. Jiang, M.-C. Hu, S.-N. Li, Y. Chen, *J. Mater. Chem. A* **2021**, *9*, 3297.
[23] X. Zou, Y. Liu, G.-D. Li, Y. Wu, D.-P. Liu, W. Li, H.-W. Li, D. Wang, Y. Zhang, X. Zou, *Adv. Mater.* **2017**, *29*, 1700404.
[24] X. Jia, Y. Zhao, G. Chen, L. Shang, R. Shi, X. Kang, G. I. N. Waterhouse, L.-Z. Wu, C.-H. Tung, T. Zhang, *Adv. Energy Mater.* **2016**, *6*.
[25] J. Liu, D. Zhu, T. Ling, A. Vasileff, S.-Z. Qiao, *Nano Energy* **2017**, *40*, 264.
[26] B. Qiu, L. Cai, Y. Wang, Z. Lin, Y. Zuo, M. Wang, Y. Chai, *Adv. Funct. Mater.* **2018**, *28*, 1706008.
[27] G. Liu, X. Gao, K. Wang, D. He, J. Li, *Int. J. Hydrogen Energy* **2016**, *41*, 17976.
[28] H. Liu, G. Xia, R. Zhang, P. Jiang, J. Chen, Q. Chen, *RSC Adv.* **2017**, *7*, 3686.
[29] X. Jia, Y. Zhao, G. Chen, L. Shang, R. Shi, X. Kang, G. I. N. Waterhouse, L.-Z. Wu, C.-H. Tung, T. Zhang, *Adv. Energy Mater.* **2016**, *6*, 1502585.
[30] P. W. Menezes, C. Walter, J. N. Hausmann, R. Beltrán-Suito, C. Schlesiger, S. Praetz, V. Yu. Verchenko, A. V. Shevelkov, M. Driess, *Angew. Chem. Int. Ed.* **2019**, *58*, 16569.
[31] K. Xu, F. Song, J. Gu, X. Xu, Z. Liu, X. Hu, *J. Mater. Chem. A* **2018**, *6*, 14240.
[32] S. Yagi, I. Yamada, H. Tsukasaki, A. Seno, M. Murakami, H. Fujii, H. Chen, N. Umezawa, H. Abe, N. Nishiyama, S. Mori, *Nat. Commun.* **2015**, *6*, 8249.
[33] G. Hai, X. Jia, K. Zhang, X. Liu, Z. Wu, G. Wang, *Nano Energy* **2018**, *44*, 345.
[34] M. Gong, Y. Li, H. Wang, Y. Liang, J. Z. Wu, J. Zhou, J. Wang, T. Regier, F. Wei, H. Dai, *J. Am. Chem. Soc.* **2013**, *135*, 8452.
[35] S. Zhao, Y. Wang, J. Dong, C.-T. He, H. Yin, P. An, K. Zhao, X. Zhang, C. Gao, L. Zhang, J. Lv, J. Wang, J. Zhang, A. M. Khattak, N. A. Khan, Z. Wei, J. Zhang, S. Liu, H. Zhao, Z. Tang, *Nat. Energy* **2016**, *1*, 16184.

[36] Q. Xiong, Y. Wang, P.-F. Liu, L.-R. Zheng, G. Wang, H.-G. Yang, P.-K. Wong, H. Zhang, H. Zhao, *Adv. Mater.* **2018**, *30*, 1801450.
[37] J. Zhang, T. Wang, D. Pohl, B. Rellinghaus, R. Dong, S. Liu, X. Zhuang, X. Feng, *Angew. Chem. Int. Ed.* **2016**, *128*, 6814.
[38] H. Li, S. Chen, X. Jia, B. Xu, H. Lin, H. Yang, L. Song, X. Wang, *Nat. Commun.* **2017**, *8*, 15377.
[39] X. Xu, F. Song, X. Hu, *Nat. Commun.* **2016**, *7*, 12324.
[40] T. Wang, G. Nam, Y. Jin, X. Wang, P. Ren, M. G. Kim, J. Liang, X. Wen, H. Jang, J. Han, Y. Huang, Q. Li, J. Cho, *Adv. Mater.* **2018**, *30*, 1800757.
[41] Q. Cao, J. Yu, K. Yuan, M. Zhong, J.-J. Delaunay, *ACS Appl. Mater. Interfaces* **2017**, *9*, 19507.
[42] X.-D. Wang, Y. Cao, Y. Teng, H.-Y. Chen, Y.-F. Xu, D.-B. Kuang, *ACS Appl. Mater. Interfaces* **2017**, *9*, 32812.
[43] G. Ou, P. Fan, H. Zhang, K. Huang, C. Yang, W. Yu, H. Wei, M. Zhong, H. Wu, Y. Li, *Nano Energy* **2017**, *35*, 207.
[44] K. Wang, H. Du, S. He, L. Liu, K. Yang, J. Sun, Y. Liu, Z. Du, L. Xie, W. Ai, W. Huang, *Adv. Mater.* **2021**, *33*, 2005587.
[45] T. Liu, J. Mou, Z. Wu, C. Lv, J. Huang, M. Liu, *Adv. Funct. Mater.* **2020**, *30*, 2003407.
[46] J. He, X. Zhou, P. Xu, J. Sun, *Nano Energy* **2021**, *80*, 105540.
